# Indirect violence exposure and mental health symptoms among an urban public-school population: Prevalence and correlates

**DOI:** 10.1371/journal.pone.0224499

**Published:** 2019-11-27

**Authors:** Erica L. Gollub, Jakevia Green, Lisa Richardson, Ilyssa Kaplan, Denese Shervington

**Affiliations:** 1 Health Science Program, College of Health Professions, Pace University, Pleasantville, NY, United States of America; 2 Institute of Women & Ethnic Studies, New Orleans, LA, United States of America; 3 Department of Psychology, Pace University, New York, NY, United States of America; 4 Charles R. Drew School of Medicine and Science, Psychiatry and Behavioral Sciences, Los Angeles, CA, United States of America; Stellenbosch University, SOUTH AFRICA

## Abstract

Available literature identifies the need for a deeper understanding of the role of gender, age and socioeconomic status in children’s exposure to violence and associations with mental health (MH) outcomes. The 1548 participants for this study were enrolled from 28 public charter schools and 9 community-based settings; youth were administered a screener that assessed exposure to traumatic events and symptoms of post-traumatic stress disorder (PTSD) and depression. Respondents reported extremely high levels of exposure to indirect violence: 41.7% witnessed shooting/stabbing/beating; 18.3% witnessed murder; and 53.8% experienced the murder of someone close. Frequency of adverse MH outcomes was high: 21.2% screened positive for depression; 45.7% for lifetime PTSD; and 26.9% for current PTSD. More males than females reported witnessing shooting/stabbing/beating (*p =* .04); females more often reported experiencing the murder of someone close (*p =* .001). Indirect violence exposure generally increased with age. Youth attending schools with ≥90% free/reduced lunch participation (FRLP) showed significantly higher levels of violence exposure compared to youth in schools with <90% FRLP. Females endorsed significantly higher levels of depression (21.4% vs. 9.7%), and lifetime (53.9% vs. 34.9%) and current (32.5% vs. 19.6%) PTSD, compared with males (*p <* .0001, all comparisons). Female sex (aOR = 2.6), FRLP (aOR = 1.4 for ≥90% vs. <90%) and the number of different indirect violence exposures (aORs from 1.3 to 10.4), were significantly associated with a positive screen for any adverse MH outcome. Our data add important insights into gender heterogeneity of viewed violence, mental health symptoms, and their association—all of which are critical to guiding effective intervention efforts.

## Introduction

Large proportions of children in the United States (US) are subject to violence within their homes, schools, and communities [1, 2]. Estimates of prevalence of exposure to violence are variable, ranging from 50–75% in recent work [3, 4], up from 20–50% overall in older studies [5]. In the 2008 National Survey of Children’s Exposure to Violence, a nationally representative sample of US children ages 0–17 years, 60.6% reported at least one direct victimization and 25.3% reported indirect victimization, in the last year [2]. In a 2013 update [6], the reported proportions of witnessed violence were only slightly lower (22.4%). In a recent U.S. Census Bureau study of urban adolescents ages 12–17 years, 55% reported lifetime experience of community violence [7]. Estimates are higher when considering studies focused on urban adolescents. In a 9-year longitudinal study of 6,000 children in Chicago, by 16 years of age, 85% of youth reported witnessing at least one of nine violent acts over a one-year period, and 30% reported being the victim of at least one of five violent acts [8]. In an adolescent sample aged 11–14 years from a Midwest urban school, 75% of youth witnessed hearing or seeing gunfire in their home or neighborhood [9].

Urban-residing African-American youth in particular are at very high risk of exposure to violence. In a study of African American adolescents from economically disadvantaged communities, 95% of youth endorsed exposures to more than one violent event, 30% reported hearing gunshots in the community, 65% had seen someone being threatened by a weapon, and 54% reported that someone close to them was shot or attacked [8]. In a recent cross-sectional study of younger children (aged 5–15 years) from the New Orleans area, all children reported physical aggression, and over one third (35%) reported witnessing community violence or had seen a friend or family member get hurt or mistreated [10].

The study of indirect violence exposures among youth–as distinct from all types of violence exposures—is important for a number of reasons. Prior research has suggested that viewed violence is an independent risk factor for adverse psychological sequelae [1, 8]. As well, a better understanding of the extent and nature of viewed violence might inform our appreciation of the dynamics of direct victimization on youth development. Further, national survey data show the prevalence of witnessed violence among adolescents to be greater than direct victimization [11]. Thus, although the per-exposure risk of psychological distress from direct violence may be higher, the population impact of larger numbers of youth witnessing violence or knowing someone who has been a victim of violence merits an exclusive focus on indirect violence. Finally, confidential surveys of youth regarding indirect violence exposure and perpetration have been considered as valid approaches to measure trends in youth violence for decades [12].

### Associations of violence exposure with post-traumatic stress disorder and major depressive disorder symptoms

Recent reviews of published evidence have found a moderate relationship between hearing about, witnessing and victimization of community violence. In these studies authors found hearing about and witnessing violence predicted PTSD symptoms to the same extent as victimization, leading to the consideration of the theory of “collective traumatization” to interpret this finding—a feeling that violence pervades the neighborhood and no one is safe [13]. A nationally representative sample of over 4000 adolescents aged 12–17 years surveyed on sexual and physical assaults and witnessing violence found interpersonal violence of all kinds was associated with greater than a doubling of risk for comorbid diagnoses PTSD and MDD among youth [14]. Importantly, numerous authors studying PTSD symptomatology in children and adolescents exposed to violence repeatedly and frequently note that symptoms go misdiagnosed or untreated especially in ethnic minority populations [15–17].

The association between violence exposure and depression alone (non-comorbid) is less straightforward, based on available data. Some researchers suggest that youth chronically exposed to community violence may become desensitized and suppress feelings of sadness or anxiety [13, 18], thereby explaining why MDD symptoms do not show a consistent rise with increased violence exposure. Gaylord-Harden et al. [19] point out that African American youth may be less likely to exhibit affective symptoms associated with dysphoric mood [20]. For African American youth living in high-crime communities, expressions of sadness or low self-esteem may increase vulnerability to direct victimization [20, 21]. Rather than becoming desensitized to violence, youth may be suppressing depressive symptoms to facilitate their ability to navigate dangerous neighborhoods [22]. Considerably more work remains to be done to further elucidate how and when symptoms manifest for these key adverse outcomes, which account for a significant portion of MH conditions in children and adolescents. Given the widespread exposure of children to indirect violence, additional research is needed to understand its exclusive impact on mental health, as distinct from youth exposure to direct violence.

### Challenges: Definition of exposure

The literature points to a clear relationship between childhood exposures to violence and a variety of health and social challenges that can be predicted by these experiences throughout the life course, particularly during the childhood years [23]; nevertheless, study of these exposures and their impact has been hampered by a number of limitations. Researchers and clinicians lack a common language to speak about findings, prevention, and intervention measures related to violence experienced in the home, school, and/or community, making it difficult to compare the occurrence of multiple kinds of violence and specific effects of exposures across different settings [23]. According to Kracke and Hahn [24], “exposure” encompasses an individual’s involvement or experience with both direct violence (which is targeted at the child) and indirect violence (which are acts that a child witnesses) and includes levels of traumatic experiences ranging from single, acute acts to repeated chronic ones, as shown in multiple works [18, 25, 26]. Some work has contrasted three types of violence: victimization, witnessing, and “hearing about” or vicarious exposure [13, 25], finding that negative MH outcomes frequently increase linearly with physical proximity (suggesting vicarious exposure would carry the lowest risk of negative outcomes). Other studies, however, often merge both direct violence and indirect violence when reporting findings, making it difficult to delineate the effects of each separately.

### Associations of indirect violence exposure with key demographic variables

Understanding disparities in witnessed violence and MH outcomes across key demographic correlates is essential for tailoring prevention and intervention programs aimed at specific subgroups [13].

#### Gender

A large review of data on gender differences in urban youth indicated that males were more likely than females to be victims and witnesses to violent acts [25]. Two more recent studies on 5^th^ graders in Birmingham, AL [4] and randomly selected youth in Chicago [8] documented greater violence exposure among males, which supports findings that males have higher levels of engagement in risk-taking behaviors [27]. In related findings from smaller and/or more select samples (medical and psychiatric patients; youth referred for MH care), males have reported more victimizations and perpetrations while females were more likely to report higher rates of witnessing violence, including knowing of a victimization of a close other [28–30].

Recent reviews of the moderating effect of gender on the relationship of violence exposure to MH symptoms decry the scarcity of studies and lack of consensus in available data [13, 31, 32]. Some findings point to distinct pathways for girls’ responses to witnessed violence. In a sample of 306 youth receiving outpatient psychiatric services in a large city [33], girls who heard stories of violence against known victims were more likely to develop adverse MH outcomes compared with boys; those who witnessed acts of violence against people they knew were more likely to develop externalizing MH outcomes. The researchers introduced the “cost of caring” hypothesis, advancing the idea that girls are more likely to be vulnerable to distress when it directly affects close others in their social network—a notion of “vicarious trauma” [33, 34].

Among a sample of predominantly African-American youth aged 7–18 years, Fitzpatrick and Boldizar [35] found a strong association between exposure to violence (both witnessing and direct victimizations) and self-reported PTSD symptomatology that was more pronounced in females. Zona and Milan followed a random sample of children (n = 615) in a longitudinal study of 80 Chicago neighborhoods and found girls who witnessed violence were no more likely than boys to report PTSD symptoms [8], but were more likely to report dissociative symptoms. A greater dissociative response to exposure to violence among girls is consistent with gender-specific stress responses and pathways to trauma-related PTSD and psychopathology [36–38].

#### Age

Large national surveys have found that the frequency of witnessed community violence increases with age; family violence exposure showed no age gradient [39]. Studies on more select populations also support the conclusion that witnessed violence increases with age. In a 2-year longitudinal study conducted by Weist, Acosta, and Youngstrom [30], researchers administered the Exposure to Violence Screening Measure to urban youths, examining prior exposures to violence (robbery, assaults, shootings, stabbings) and found that exposure increased with age. Numerous researchers [4, 10, 19, 40, 41] have discussed how desensitization impacts the way youth generally react to experiences and adaptive mechanisms to continuous violence exposure and develop adaptive mechanisms to continuous violence exposure that may downplay the self-reported symptoms of mental distress [42].

#### Socio-economic status (SES) as a modifier of the MH impact of indirect violence

US studies seeking to elucidate the separate effects of SES (as distinct from race) as modifiers of the impact of violence on MH have been few and challenging due to the overrepresentation of African-American youth living in impoverished, higher-violence areas. Earlier research indicated children and adolescents of lower SES from larger urban areas are at greater risk for victimization than those with larger family incomes [5]. Use of an indicator such as family income has been limited in other work.

#### Study rationale

Numerous gaps remain in our understanding of the correlates of violence exposure as well as MH sequelae of these exposures on youth. Youth responses have often been provided via proxy reporting, possibly resulting in an underestimate of violence exposures [43]. Studies have often targeted a general US population, or a very high-risk group such as referred youth; few studies involve an unselected sample of school-based youth. In measurement, indirect violence exposures have often been mixed with direct exposures. Finally, exploration of key correlates has often been hampered by an insufficient sample size.

We report on the prevalence and correlates of indirect violence exposures, and on associations between indirect violence and adverse MH symptomatology among a large, public school population of mostly African-American youth, with a focus on addressing these gaps. In particular, by restricting our attention to indirect violence, our study aimed to: (1) describe the frequency of these outcomes as distinct from direct victimization; (2) describe associations of indirect violence with symptoms of depression and PTSD; (3) explore how key demographic variables were associated with violence exposure and as possible moderators of any observed associations between indirect violence and adverse MH symptoms. We were particularly interested in the role of gender and the interplay of gender with age and SES on apparent trauma-related symptoms, given an insufficient understanding of these dynamics to date [24].

## Materials and methods

### Intervention

Believe in Youth–Louisiana (BY-LA) is a trauma-informed adolescent reproductive health intervention intended for Southeast Louisiana youth ages 11–19 years. Youth are enrolled from a variety of settings including middle and high schools, community-based organizations, after-school programs, and juvenile justice programs. BY-LA is a 12-module intervention delivered in one-hour group sessions by trained health educators. Program content is comprised of the eight modules of the evidence-based reproductive health curriculum *Making Proud Choices*! *Fourth Edition* [44] and four evidence-informed emotional wellness modules developed by the Institute of Women & Ethnic Studies (IWES). The emotional wellness modules are designed to promote positive stress management and coping skills and are intended to help youth recognize how mental and emotional well-being impact risk behavior and sexual decision-making.

### Participants and procedure

The 1548 BY-LA participants for this study were enrolled from a convenience/volunteer sample of 28 public charter schools and 9 community-based settings. An Emotional Wellness Screener (EWS) developed by IWES was administered during the program supported by an IWES licensed social worker (LMSW). A parent/guardian was required to indicate active consent for all youth participants prior to enrollment in the program including the EWS. Before the emotional wellness modules were implemented to students, health educators proctored the EWS, a 31-item questionnaire adapted from the Diagnostic Statistical Manual of Mental Disorders, Fourth Edition, (DSM-IV) clinical diagnostic criteria [17] and national youth surveys [11, 45], to assess symptomatology of post-traumatic stress disorder (PTSD) and depression, suicidal ideation, exposures to traumatic events, and worries about basic needs. Every student who indicated suicidal ideation received a private, in-person psychosocial assessment from an on-site counselor, social worker, or other MH professional and each case was discussed with board-certified psychiatrist who directs the BY-LA program. Site-specific, aggregate results of the EWS data were provided to each site in follow-up meetings concluding implementation.

### IRB approval

IWES utilizes a community-based institutional review board (IRB) registered with the Office for Human Research Protections of the U.S. Department of Health & Human Services. The BY-LA intervention, including the EWS, was reviewed and approved by the IRB in October 2015. At the time of review and approval, the IRB was comprised of professionals of varying backgrounds and expertise—sociology, social work, public health, and nursing—and also included a recent high school graduate from the community to represent the perspective of intervention participants.

### Measures

#### Demographic characteristics

Participants’ demographics were collected at the time of enrollment with the parental consent form for participation in the BY-LA program. Age, race, ethnicity, gender, grade, and enrollment site (i.e. school, community-based organization, or program) were among demographic measures collected. For this study both continuous and categorical scaling for age (age group defined as ≤14 years or >14 years) and grade (grade group defined as <9^th^ grade or ≥9^th^ grade) were used.

#### Mental health disorder symptomatology and indirect violence exposure

Extracts of the Emotional Wellness Screener were analyzed in this study to assess participant symptomatology for PTSD and depression as well as experiences of indirect violence exposure (S1 Table).

#### Symptomatology of PTSD and depression

The first two sections, Items 1–17, of the Emotional Wellness Screener assess symptomatology of PTSD and depression, respectively. Items 1–8 of the EWS screen for symptoms of PTSD due to one or more unspecified traumatic events experienced at any point during the participant’s lifetime. The assessed symptoms align with the DSM-IV diagnostic criteria for PTSD—re-experiencing the trauma(s), persistent avoidance of stimuli associated with the trauma(s) and numbing of general responsiveness, and increased arousal due to the traumas. For this study, individuals answering “Yes” to experiencing four or more items for as long as one month or more after the event(s) are considered to endorse symptomology of PTSD. Item 9 assesses whether or not four or more symptoms of PTSD were experienced within the 30 days preceding the survey date. Items 10–17 of the EWS assess symptoms of depression within the 14 days preceding the survey date and follow DSM-IV scoring criteria. After summing the total number of “Yes” responses for this section, a score of five or more with one of the following patterns indicates symptomatology of depression: 1) “Yes” to Item 10 (feeling sad or blue) *without* Item 11 (having lost interest in things and/or people that were enjoyed) and “Yes” to four additional symptoms, 2) “Yes” to Item 11 *without* Item 10 and “Yes” to four additional symptoms, or 3) “Yes” to both Items 10 and 11 as well as three additional symptoms. For a positive screen of depression, Item 10 and/or 11 must be positive, plus 5 other symptoms.

#### Indirect violence exposure

Items 18–21 query participants for violence witnessed personally, including violence against a parent perpetrated by another parent or a parent’s significant other; against a sibling perpetrated by a parent; witnessing a shooting, stabbing, or beating (excluding games or other media entertainment); and murder (excluding games or other media entertainment). Item 22 assesses whether or not the participant has experienced the murder of someone close, such as a friend, neighbor, classmate, or relative.

### Statistical methods

Data from this survey were first entered into Social Solutions Efforts to Outcomes^™^ software, exported to Microsoft Excel, and converted to SAS® data files for analysis. All analyses were conducted using SAS® Studio of SAS® University Edition. Univariate analyses were conducted to describe the demographic characteristics of the sample as well as the prevalence of indirect violence exposures and adverse MH symptomatology. Chi-squared tests were conducted to test for gender differences across all variables of interest; differences between age groups and across levels of free/reduced lunch participation (dichotomized as <90% and ≥90%) were also explored with the same approach. We generated Cramer’s V statistics and used conventional thresholds to determine statistical significance (p< 0.05). The results of the univariate analyses were used to guide multivariate regression analyses which explored associations of indirect violence exposures with the presence of adverse MH symptomatology; assessed associations of age, gender, and FRLP with adverse MH symptomatology and indirect violence exposure, respectively; and assessed any interaction between FRLP and gender in the presence of adverse MH symptomatology. The generated odds ratios (ORs) and corresponding confidence intervals (CIs) were evaluated.

#### Imputation

The proportion of missing data was low (< 10%) for the main variables to be analyzed. We elected to use imputation for missing data to reduce magnifying their effect in our planned analyses which involved bivariate modeling and various levels of stratification. Values for age group were imputed by reviewing the range of ages within each grade level represented among the sample and assigning the majority age group to the subjects with missing values (e.g. only one of 154 subjects in sixth grade was older than 14 years therefore sixth grade students with missing age group values were imputed as ≤14 years). Missing values for age group were not imputed for those in grade levels in which age variability was high. Fifty-nine of the 175 cases of missing age were imputed, bringing missing age data from 6.8% to 4.5%. Gender was not reported for 149 cases, 5.8% of the total sample. To impute values, two raters independently assigned gender based on participant name, and a third rater rated subjects for which there was disagreement between the first two raters. Grade level data was missing for 221 cases, 8.6% of the total sample. Discrete grade level (6 through 12) could be determined in cases in which implementation of particular cohorts was known to be conducted with a single grade level (i.e., missing values for grade were imputed based on the grade level of participants in the same cohort). Imputation reduced the proportion missing to 4.5%. Values for grade group were imputed where possible from school type attended (ie. high school vs. middle school).

#### Computed measures

As a proxy for economic disadvantage, a measure of the proportion of students eligible for free and reduced lunch (“free and reduced lunch participation” or FRLP) across schools attended by BY-LA participants was constructed using information provided by *The New Orleans Parents’ Guide to Public Schools* [46]. In addition to analyzing distinct indirect violence exposures, related measures were coupled to add a level of categorization to violence exposure. “Family violence” comprised witnessing violence against a parent and/or sibling, and “community violence” comprised witnessing shooting/stabbing/beating and/or murder, with no specification of locale or the subject’s relation to the victim. MH symptomatology was analyzed in the following variations: 1) *Any MH outcome* (positive screening for presumptive PTSD and/or depression), 2) *Depression and PTSD* (comorbidity of PTSD and depression symptomology), 3) *Depression-only* (positive screening for depression only), and 4) *PTSD-only* (positive screening for PTSD only).

## Results and discussion

**Fig 1:**
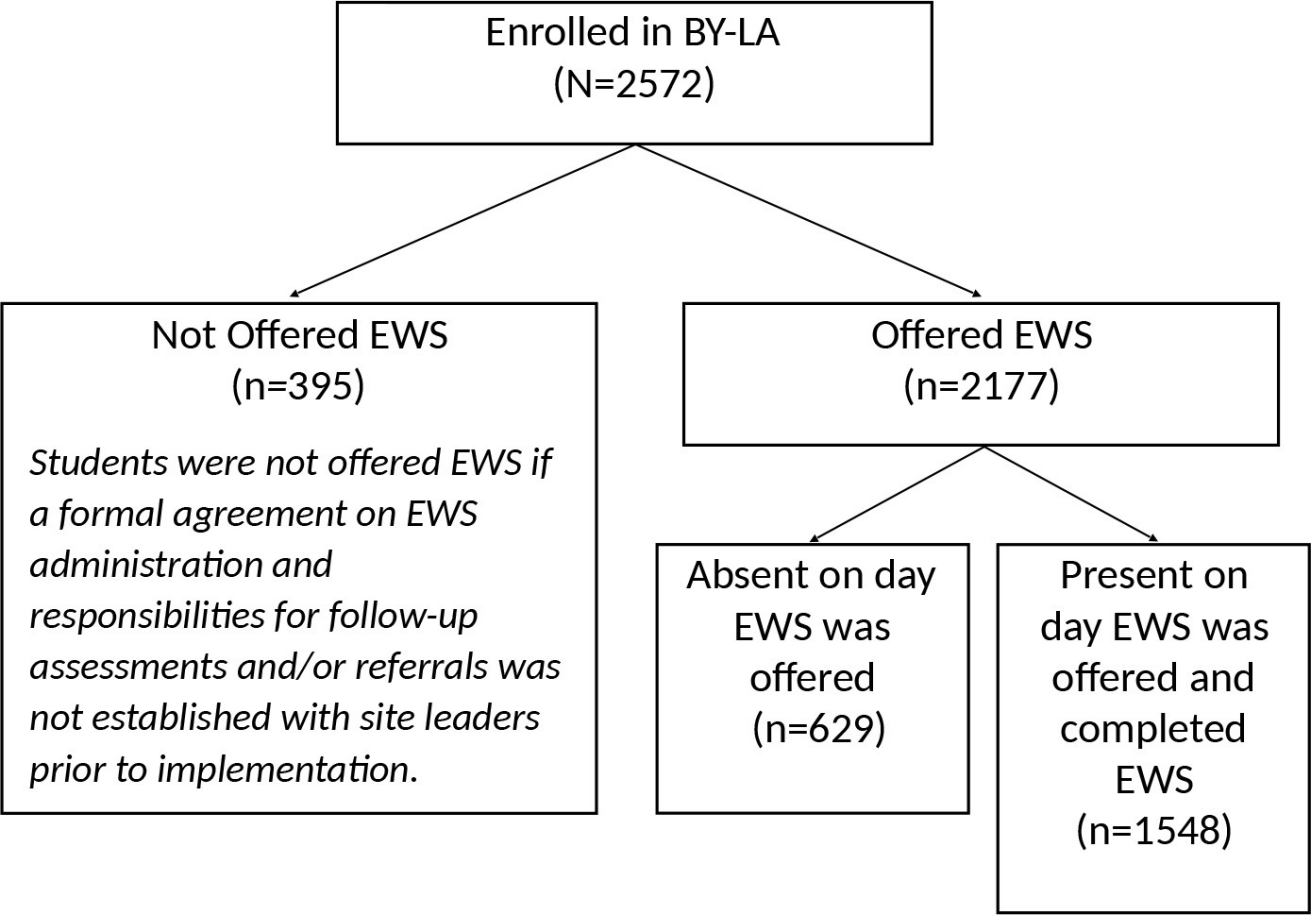
Consort diagram of BY-LA emotional wellness screener (EWS) completion.

From January 2016 to May 2017, 2572 youth were eligible to complete the EWS; 629 (24.4%) were absent on the day the survey was administered, and 395 (15% of those eligible) were not offered the survey (Fig 1). A total of 1548 participants were administered and completed the EWS (mean age 13.5 years (range 11–19); 56.7% female, and 43.3% male; see Table 1). Most youth (93.0%) identified as African American, and 8.1% identified as Hispanic/Latino. Youth in grades 7 (25.3%), 8 (28.8%), and 9 (25.4%) comprised the majority of the sample. Participating schools (n = 28) had a high proportion of students eligible for participation in free/reduced lunch (FRLP; mean 90.1%; median 91.7% range 66.8–96.3%).

**Table 1:** Descriptive statistics of IWES believe in youth–louisiana (BY-LA) participants (N = 1548).

DEMOGRAPHICS		
Measure	*n*	%
Gender	1547	
Female	877	56.7
Male	670	43.3
Race	1426	
Black/African American	1326	93.0
White	44	3.1
More than one race	38	2.7
Asian	12	0.8
American Indian/Alaska Native	6	0.4
Ethnicity	903	
Hispanic/Latino	830	91.9
Not Hispanic/Latino	73	8.1
Age (years)	1478	
≤14	1136	76.9
>14	342	23.1
Grade	1533	
<9^th^	976	63.7
≥9^th^	557	36.3
% Students eligible for free/reduced lunch of attended school (Mean 88,2, SD 8.9; Range: 66.8–96.3)	1149	
<90	792	68.9
≥90	357	31.1
**INDIRECT VIOLENCE EXPOSURE**		
Witnessed violence against parent	1519	
Yes	462	29.8
No	1057	68.3
Witnessed violence against sibling	1523	
Yes	252	16.3
No	1271	82.1
Witnessed shooting/stabbing/beating	1531	
Yes	645	41.7
No	886	57.2
Witnessed murder	1523	
Yes	283	18.3
No	1240	80.1
Experienced murder of someone close	1525	
Yes	832	53.8
No	693	44.8
**ADVERSE MENTAL HEALTH SYMPTOMATOLOGY**		
Positive screen for depression	1546	
Yes	328	21.2
No	1218	78.7
Positive screening for lifetime PTSD	1544	
Yes	708	45.7
No	836	54.0
Positive screening for current PTSD	1544	
Yes	416	26.9
No	1128	72.9

### Univariate and bivariate results

#### Overall

EWS respondents reported extremely high levels of lifetime exposure to violence: 29.8% witnessed violence against a parent; 41.7% witnessed a shooting/stabbing/beating; 18.3% witnessed a murder; and 53.8% experienced the murder of someone close (Table 1). Frequency of symptoms for adverse MH outcomes was high: 21.2% screened positive for symptoms of current depression; 45.7% for lifetime symptoms of PTSD; and 26.9% for current symptoms of PTSD. The refusal rate was less than 2% for all items.

#### Gender

There were a number of gender differences in exposure to violence and reported MH symptomatology. More males (45.2%) than females (39.9%) reported witnessing a shooting/stabbing/beating (*p =* .04), yet females more often reported experiencing the murder of someone close (males 49.8% vs females 58.2%; *p =* .001; Table 2). Males more often than females indicated that they had witnessed a murder, though this difference did not attain statistical significance. Family violence exposures were reported at virtually equivalent rates in boys and girls—approximately 30% for violence against a parent, and 16% for violence against a sibling. Females endorsed significantly higher levels of symptoms of current depression (21.4% vs. 9.7%), and lifetime (53.9% vs. 34.9%) and current (32.5% vs. 19.6%) PTSD symptomatology, compared with males (*p <* .0001 all comparisons; Table 2).

**Table 2:** Indirect exposure to violence and adverse mental health symptomatology by gender (N = 1548).

Measure	Males n (%)	Females n (%)	Cramer’s V	*p*
Witnessed parent get pushed, slapped, hit, punched, or beaten by another parent or parent’s partner	191 (29.16)	271 (31.40)	-0.0241	0.3471
Witnessed sibling get pushed, slapped, hit, punched, or beaten by parent (not including spanking)	114 (17.19)	138 (16.07)	0.0151	0.5567
Witnessed shooting/stabbing/beating	299 (45.17)	346 (39.86)	0.0532	0.0374
Witnessed murder	134 (20.24)	149 (17.33)	0.0372	0.1472
Experienced the murder of someone close	326 (49.77)	506 (58.23)	-0.0841	0.0010
Positive screening for depression	85 (12.72)	243 (27.71)	-0.1815	<0.0001
Positive screening for lifetime PTSD	234 (34.14)	473 (53.93)	-0.1891	<0.0001
Positive screening for current PTSD	131 (19.67)	285 (32.50)	-0.1447	<0.0001

#### Age and grade level

The proportion of youth indicating any history of indirect violence exposure generally increased with age (and grade level, see S2 Table), as did the proportions for each specific type of exposure (Table 3). At younger ages (≤14 years), 25% of the youth reported having had none of the indirect exposures described in the EWS; at older ages (>14 years), this proportion was lower, at 19% (*p =* .018). In addition, among those who reported any indirect violence exposure experienced over their lifetime, the mean number of different exposure types was 1.54 for youth ≤14 years, and 1.78 for youth >14 years. Positive screens for current and lifetime PTSD symptomatology, unlike those for current depression, were most frequent at younger ages and grades.

**Table 3:** Indirect exposure to violence and adverse mental health symptomatology by age group (N = 1548).

Measure	≤14 years n (%)	>14 years n (%)	Cramer’s V	*p*
Witnessed parent get pushed, slapped, hit, punched, or beaten by another parent or parent’s partner	328 (29.42)	117 (34.82)	-0.0494	0.0597
Witnessed sibling get pushed, slapped, hit, punched, or beaten by parent (not including spanking)	175 (15.68)	60 (17.70)	-0.0232	0.3765
Witnessed shooting/stabbing/beating	449 (39.95)	158 (46.64)	-0.0570	0.0291
Witnessed murder	179 (16.04)	88 (26.04)	-0.1091	<0.0001
Experienced the murder of someone close	611 (54.60)	186 (55.03)	-0.0036	0.8900
Positive screening for depression	231 (20.35)	80 (23.46)	-0.0321	0.2172
Positive screening for lifetime PTSD	534 (47.09)	142 (41.76)	0.0464	0.0839
Positive screening for current PTSD	310 (27.34)	86 (25.29)	0.0204	0.4561

#### School free/reduced lunch participation

Increasing level of FRLP (categorized as ≥90% highest vs. <90% lower) of schools was associated with a consistent rise across all indirect violence exposures, as well as with adverse MH symptomatology in the frequency of youth reporting these outcomes; most of these differences were statistically significant. When stratifying by gender, it was apparent that statistically significant increases in violence exposures with increased FRLP were seen considerably more often in males than in females; but significant changes in MH symptom frequency with increased FRLP occurred more often in females than in males (Table 4). When looking at the absolute frequency of youth reporting violence exposure, females in schools with a lower FRLP showed a trend toward higher exposure proportions than males within that FRLP category. At higher FRLP schools, however, the trend was reversed, with males reporting somewhat higher exposure for most types of violence with the exception of experiencing the murder of someone close. Given small cell sizes, these differences were likely not statistically significant.

**Table 4:** Indirect exposure to violence and adverse mental health symptomatology among males and females, by percentage of student free/reduced lunch participation (FRLP) of attended school (N = 1548).

Measure	Males		Cramer’s V	*p*	Females		Cramer’s V	*p*
	≥90% FRLP n (%)	<90% FRLP n (%)			≥90% FRLP n (%)	<90% FRLP n (%)		
Witnessed parent get pushed, slapped, hit, punched, or beaten by another parent or parent’s partner	103 (34.22)	29 (17.58)	0.1766	0.0001	154 (32.35)	54 (30.17)	0.0209	0.5924
Witnessed sibling get pushed, slapped, hit, punched, or beaten by parent (not including spanking)	58 (19.27)	19 (11.05)	0.1071	0.0198	87 (18.24)	25 (13.97)	0.0506	0.1952
Witnessed shooting/stabbing/beating	134 (44.37)	55 (31.98)	0.0085	0.0081	201 (41.88)	66 (36.46)	0.2065	0.2061
Witnessed murder	67 (22.19)	24 (13.95)	0.0295	0.0287	83 (17.44)	30 (16.57)	0.0102	0.7936
Experienced the murder of someone close	161 (54.21)	69 (40.59)	0.1311	0.0046	289 (60.21)	92 (50.83)	0.0846	0.0295
Positive screening for depression	41 (13.49)	14 (8.05)	0.0820	0.0729	145 (29.77)	39 (21.55)	0.0819	0.0344
Positive screening for lifetime PTSD	107 (35.31)	53 (30.64)	0.0490	0.2987	286 (58.73)	89 (49.17)	0.0856	0.0270
Positive screening for current PTSD	59 (19.47)	29 (16.76)	0.0345	0.4640	60 (33.15)	169 (34.70)	0.0145	0.7070

#### Gender and age

In stratified analyses by age category (≤14 years vs. >14 years), additional gender differences emerged; moving from younger to older ages, more boys than girls witnessed a shooting/stabbing/beating (*p =* .048) and witnessed a murder (*p =* .02); and differences between boys and girls in experiencing the murder of someone close, though substantial, were no longer statistically significant though lower frequencies may have accounted for this (Table 5). Over all ages, 58% of girls and 37% of boys endorsed symptoms of depression and/or PTSD. More girls (24%) than boys (10%) endorsed symptoms of both conditions (i.e. co-morbid).

**Table 5:** Indirect exposure to violence and adverse mental health symptomatology among younger (≤14 years) and older (>14 years) youth, by gender (N = 1548).

Measure	≤14 years		Cramer’s V	*p*	>14 years		Cramer’s V	*p*
	Males n (%)	Females n (%)			Males n (%)	Females n (%)		
Witnessed parent get pushed, slapped, hit, punched, or beaten by another parent or parent’s partner	131 (28.67)	197 (29.98)	-0.0142	0.6346	54 (33.33)	63 (36.21)	-0.0301	0.5806
Witnessed sibling get pushed, slapped, hit, punched, or beaten by parent (not including spanking)	75 (16.30)	100 (15.27)	0.0140	0.6393	29 (17.47)	31 (17.92)	-0.0059	0.9137
Witnessed shooting/stabbing/beating	194 (42.08)	255 (38.52)	0.0358	0.2305	86 (52.12)	72 (41.38)	0.1076	0.0475
Witnessed murder	76 (16.49)	103 (15.75)	0.0099	0.7414	52 (31.71)	36 (20.69)	0.1255	0.0211
Experienced the murder of someone close	224 (49.23)	387 (58.37)	-0.0902	0.0026	85 (51.83)	101 (58.05)	-0.0625	0.2509
Positive screening for depression	49 (10.54)	182 (27.20)	-0.1761	<0.0001	30 (18.07)	50 (28.57)	-0.1154	0.0222
Positive screening for lifetime PTSD	165 (35.56)	368 (55.01)	-0.1932	<0.0001	55 (33.33)	87 (49.71)	-0.1702	0.0022
Positive screening for current PTSD	90 (19.40)	220 (32.88)	-0.1498	<0.0001	33 (20.00)	53 (30.29)	-0.1213	0.0292

**Fig 2:**
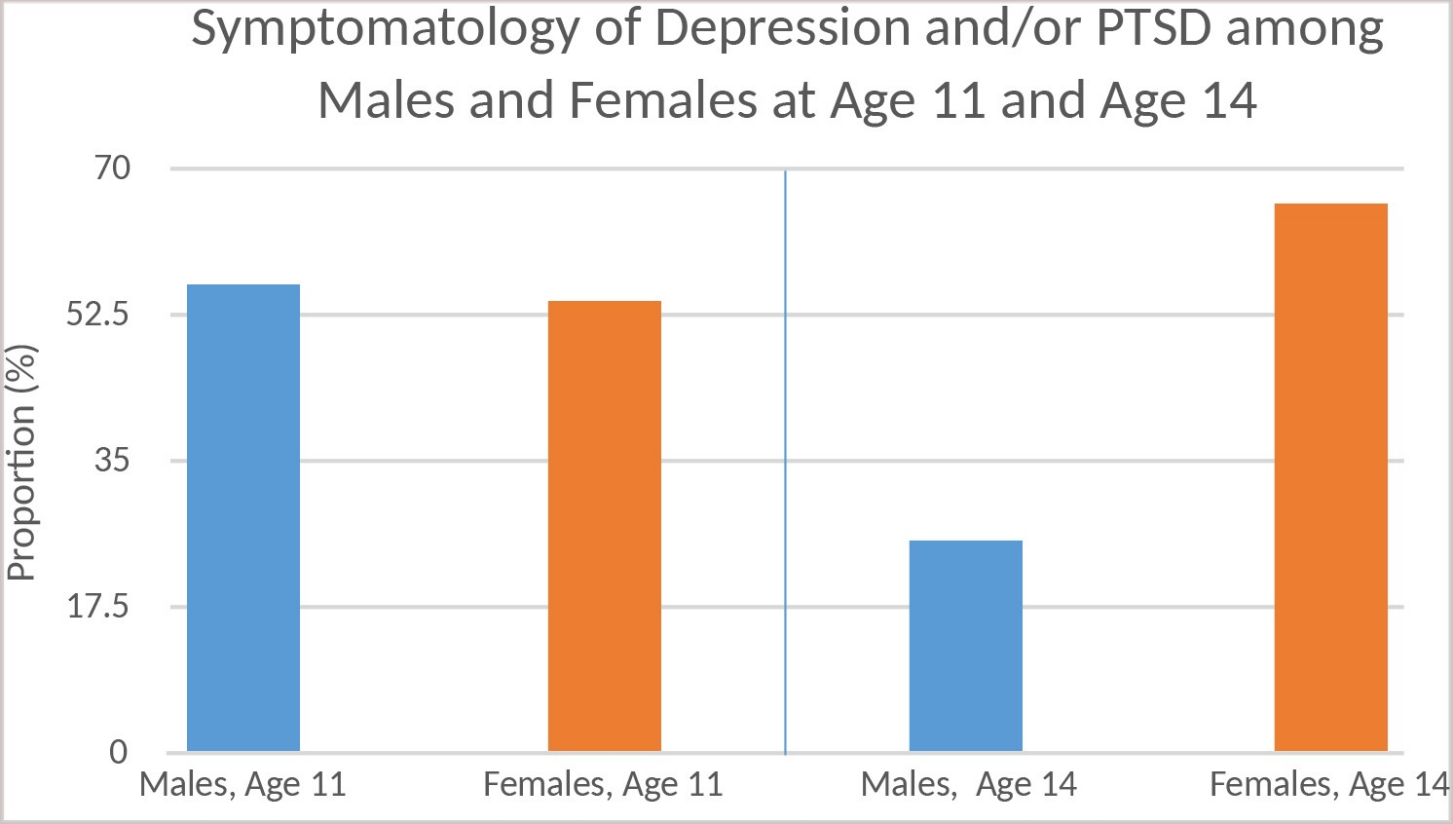
Symptomatology of depression and/or PTSD among males and females at age 11 and age 14.

When restricting attention to middle school aged youth (11–14 years), in continuous analyses of yearly age, endorsement of PTSD (lifetime and/or current) symptoms among boys exhibited a marked linear decline with increasing age, compared with a much slower decline among girls. At age 11 years, for example, 43.5% of boys and 36.8% of girls endorsed PTSD symptoms (without depression); by age 14 years, the ratio reversed to 18.9% and 28.9%, respectively. Data on depression only were too sparse for meaningful gender comparisons. At age 11 years, high proportions—56.5% of boys and 54.4% of girls—endorsed depression and/or PTSD symptomatology; by age 14 years, the striking and conventional gender-based imbalance had emerged, with these proportions at 25.7% and 64.9%, respectively (Fig 2). Due to sparse data in older age groups (especially for boys) trends after 14–15 years of age were difficult to discern.

#### Types of indirect violence exposures

The number of distinct types of indirect violence exposures was directly proportional to the percentage of youth endorsing symptoms of any MH outcome; percentages ranged from 44.0%-100% in girls and 21.5%-62.5% in boys for 0 to 5 distinct exposures (Table 6).

**Table 6:** Number of indirect exposure types and positive symptomatology for depression and/or PTSD by gender (N = 1548).

Measure	Positive symptomatology for depression and/or PTSD	
	Males n = 164	Females n = 380
Sum of reported indirect violence exposure types		
0	29 (21.48)	62 (43.97)
1	34 (28.10)	100 (51.55)
2	41 (48.81)	93 (64.58)
3	36 (52.17)	70 (72.16)
4	19 (54.29)	45 (86.54)
5	5 (62.50)	10 (100.00)

We compared two distinct sets of indirect violence exposures as independent variables: “community violence” (comprising ever witnessing shooting/stabbing/beating or murder, not specified as parent or sibling) and “family violence” (comprising ever witnessing a sibling or a parent get pushed, slapped, hit, punched, or beaten). Family violence demonstrated moderately strong and significant associations with endorsed symptoms of any MH outcome (OR = 2.6; *p <* .0001), and of both depression and PTSD (ie. comorbid; OR = 3.0, *p*< .0001). Community violence demonstrated similar results, associated with more than a doubling in the odds of reported symptoms of both PTSD and depression (OR = 2.6; *p <* .0001) and a near doubling in odds of reported symptoms of any MH outcome (OR = 1.95; *p <* .0001).

#### Multivariate analyses

Multivariable regression analyses of exposure to violence (any/none) revealed significant associations with gender and FRLP. Females had 1.4 times the odds of indirect violence exposure as males. Students at schools with higher FRLP (≥90%) had 1.8 times the odds of indirect violence exposure compared with students at schools with lower FRLP (Table 7). Increasing age was also significantly associated with violence exposure.

**Table 7:** Multivariate logistic regression model for any indirect violence exposure (Y/N) (N = 1548).

Characteristic (referent)	OR (95% CI)
Age (standardized)	1.28 (1.02–1.61)
Gender (Male)	
Female	1.43 (1.08–1.90)
Free/Reduced Lunch Participation (<90%)	
≥90%	1.86 (1.40–2.48)

When analyzing results for our combined MH variable, any MH outcome, we found significant associations for female gender (aOR = 2.60), FRLP (aOR = 1.35), and number of different indirect violence exposure types (aORs from 1.34 to 10.43; p for trend < 0.0001; Table 8). Although a formal test for an interaction between gender and FRLP when added to the multivariable model was not statistically significant (p = 0.18), gender-stratified multivariable regression analyses of correlates of MH symptoms were suggestive of an interplay between these two variables. Adjusting for age and frequency of different violence exposures, the odds of adverse MH symptomatology were increased with a high FRLP in girls (aOR 1.6; *p =* .01) but in boys there was no such significant association (aOR = 1.1; *p =* .64; Table 9).

**Table 8:** Multivariate logistic regression model for any MH outcome (N = 1548).

Characteristic (referent)	Positive symptomatology for depression and/or PTSD (n = 544)
	OR (95% CI)
Gender (Male)	
Female	2.60 (2.00–3.83)
Age (≤14)	
>14	0.93 (0.67–1.31)
Free/Reduced lunch participation of attended school (<90%)	
≥90%	1.35 (1.03–1.79)
Sum of reported indirect violence exposure types (0)	
1	1.34 (0.95–1.90)
2	2.62 (1.80–3.82)
3	3.45 (2.27–5.24)
4	5.46 (3.14–9.50)
5	10.43 (2.86–38.03)

**Table 9:** Multivariate logistic regression models for any MH Outcome, by gender (N = 1548).

Characteristic (referent)	Males	Females
	Positive screening for depression and/or PTSD (*n* = 164)	Positive screening for depression and/or PTSD (*n* = 380)
	OR (95% CI)	OR (95% CI)
Age (≤14)		
>14	0.93 (0.55–1.57)	0.99 (0.64–1.55)
Free/Reduced lunch participation of attended school (<90%)		
≥90%	1.11 (0.72–1.70)	1.63 (1.13–2.35)
Sum of reported indirect violence exposure types (0)		
1	1.40 (0.79–2.50)	1.32 (0.85–2.05)
2	3.42 (1.88–6.21)	2.21 (1.37–3.58)
3	3.91 (2.08–7.38)	3.18 (1.82–5.56)
4+	4.52 (2.15–9.51)	9.91 (4.42–23.36)

Results for the two regression analyses remained significant after applying a Bonferroni correction for multiple tests [47].

## Discussion

Data gathered in the Emotional Wellness Screener, administered to a sample of 6^th^ to 12^th^ grade students enrolled in New Orleans public schools, demonstrated extremely high levels of indirect violence that agreed with findings from other urban samples of youth [30, 32] and were substantially greater than those reported in national surveys of youth [2], underscoring the importance of focusing on specific populations for intervention efforts. Our large sample, and inclusion of both middle and high school age youth, allowed for finer analyses of age effects even in the absence of a longitudinal design, as well as age by gender analyses that rendered certain unexpected findings. The wider age distributions of some prior studies may mask the true experiences of adolescents, where MH symptoms become particularly pronounced

We uncovered clear associations of both gender and economic hardship with violence exposure as well as with MH symptoms; additionally, gender worked as a seeming moderator of the association between economic hardship and MH symptoms. Prior reviews of the relationship of violence with adolescent MH [12, 30] have pointed to the relative scarcity of work on gender as a moderator. Our work responds to this identified gap.

In our data, gender was associated with different types of violent exposures—in particular, girls’ “vicarious exposure” (i.e., knowing someone who was murdered) was considerably higher than that of boys, across both age and school-based SES categories. In univariate analyses, boys more frequently witnessed shootings, stabbings and beatings, and, to a lesser extent, murders, with considerable variation by school-based SES. The findings support a generally held view that boys experience the worst/most severe direct witnessing of violence [25, 27, 32, 48]; nevertheless, our findings on girls’ vicarious exposure [28, 30] also lend credence to the “cost of caring” hypothesis advanced by some [34, 49]. In adjusted analyses, girls had a greater overall odds of violence exposure, reflecting complex relationships among our measured variables.

We also found that the progression of MH symptoms over the middle school years worked differently for boys and girls. Endorsement of any measured MH symptoms demonstrated opposite trends for boys and girls; symptom reporting in boys decreased markedly over age 11–14 years, by more than half; girls’ endorsed symptoms over these same ages rose by more than 10 percentage points. The results for PTSD-only seem to substantiate the idea of desensitization over adolescence, proposed by several prior reports [4, 40, 41], but with a clear gender heterogeneity. Our results also provide a more precise picture of when these mechanisms begin to act. The data lend support to a recent American Psychological Association report that perceptions of masculinity—such as control and toughness—drive behavior such that minority males may suppress emotions when traumatized or hide symptoms of depression [50]. Conversely, researchers introduced the “cost of caring” hypothesis to describe increased MH symptoms among girls who are showing evidence of “vicarious trauma” and reporting adverse MH reactions when witnessing violence against people they know [33, 34]. Our gender specific analysis at all ages combined showed that boys, like girls, reported increasing adverse MH symptoms as the frequency of distinct violent exposures increased. At older ages, however, our results suggest this dose response no longer holds for boys. Among the girls, depressive symptoms rose to 65% by age 14 years.

Finally, the data suggest a remarkably consistent association of our socio-economic status indicator with indirect violence exposure and with depressive and PTSD-associated symptoms, even among this sample of youth all of whom were attending New Orleans public schools. Indeed, the free/reduced school lunch variable was the single variable associated with uniform increases across all types of indirect violence exposure and all types of adverse MH symptomatology—in contradistinction to our other main covariates, age and gender, which showed heterogeneous effects across these outcomes. These findings agree with some prior studies, most notably with a longitudinal study on 5^th^ graders indicating that children from lower income families witnessed more school and community violence [4], as well as with data from the nationwide survey of Finkelhor et al. [2] suggested greater victimization rates among children of lower socioeconomic status. Recent work tying neighborhood degradation to prevalence of violence (and the “greening hypothesis” [51]) may illuminate fruitful areas for further research, although direct measurement of the differences in the micro-environment of the schools in our sample were beyond the scope of this research.

Too, our analyses suggested important interactions of gender with age and socioeconomic disparity that require future study and elaboration. Specifically, although increased levels of our school-based economic hardship indicator were associated in adjusted analyses with greater likelihood of any MH symptom reporting overall, upon gender stratification, this effect appeared to be most pronounced among girls; economic hardship did not significantly affect the odds of adverse MH symptom reporting in boys. These results are not easily understood and there are few corresponding analyses of these variables in existing research. A recent review by Richards et al. [52] references several studies indicating that lower income African American adolescent girls have higher cumulative rates of violence witnessing and victimization [53, 54], thus it is possible that other unmeasured variables account for the difference found here, although in our data, economic hardship was associated for both boys and girls with increasing indirect violence exposure.

### Limitations

Our study had numerous limitations. The data were cross sectional, thus limiting causal inferences between violence exposure and MH symptoms. Much existing literature on these associations is also based on cross sectional data; nevertheless, there is considerable agreement with results of available longitudinal studies. Because we did not systematically survey all age eligible students, our sample may not be representative of all students in the target population; participating students may have been different in key variables measured as compared to those not surveyed. Witnessed violence and symptom reporting prevalence estimates for the sample as a whole (i.e. external validity) would have been the most vulnerable to such a bias, with the internal comparisons across covariates to be less at risk of distortion. Our sample had slightly greater female participation (56%) in comparing the overall New Orleans public school population (51% female), as well as a somewhat higher FRLP (weighted average FRLP, 88.7%) than the New Orleans schools’ overall average (84%). Our sample included proportionately greater numbers of African-American youth (93% vs. 81%, New Orleans overall), fewer whites (3.1% vs. 9%), and a comparable proportion of Hispanics (8.1% vs. 7%). If our results overestimate the overall prevalence of the main outcomes, the magnitude of the bias is small, and does not change our main conclusion that these youth experience alarming levels of witnessed violence and serious MH symptoms. Finally, our violence prevalence estimates largely agree with those from other similar samples [9, 32].

Our study included relatively few demographic/predictor variables to work with and we did not measure other potentially moderating exposures, such as victimization or exposure to other types of violence that might have an association with the outcome variables we measured and thus impact the results and conclusions. Substantial proportions of youth, especially girls, reported adverse symptoms even in the absence of any indirect violence, likely indicating other exposures that were not captured in this study. We could not distinguish between certain grouped variables (e.g. stabbings and shootings), and we did not have precise data on the site where exposure occurred (e.g. school vs. community). Some prior work [4] has found violence exposure to occur more frequently in schools versus the community. This distinction, though certainly valuable for informing targeted intervention efforts, does not substantially impact our conclusions. Finally, as with most of the existing literature on these associations, endorsement of MH symptoms does not necessarily indicate a clinical diagnosis.

Our study also has numerous strengths, as noted throughout. Our large sample of non–referred, public school students provides the basis for community and school-based intervention efforts that would have a large impact. Violence exposures and MH symptoms were self-reported, reducing error and bias compared with proxy reporting. Our MH symptom measures were validated and piloted. We included key covariates—including a (school-based) SES variable, still infrequent in existing research—and our large sample allowed for finer analyses to more fully explore gender, SES, and moderation effects. We included two types of indirect exposure: witnessing and vicarious, generating certain rich gender specific findings for further elaboration. Finally, we analyzed both student age and school grade, and validated similar findings across the two variables, thus providing substantial confidence in the results presented.

## Conclusions

Our results elaborate on prior research in important ways. Adverse MH symptom prevalence in our population of youth increased proportionately with frequency of different exposures to violence; adjusted analyses confirmed an association with these symptoms. There is no current consensus on the relative importance of frequency or intensity of violence exposure in MH consequences [3]. Our work clearly suggests that *different types* of indirect exposure to violence, whether viewed or vicarious, also moderate risk of symptom reporting.

These data contribute important evidence of the urgent need for trauma informed school-based and other community-based awareness, training, intervention, and policy initiatives that recognize the high level of indirect violence exposures of students and development of associated adverse serious MH symptoms [55]. Interventions must target the different, gendered experiences of girls and boys, with specific attention to how youth process and cope with exposure to violence. It is important to note that during the early adolescent years, while boys may underreport the impact of high exposures of witnessing violence on their MH because they are desensitized, girls report symptoms that suggest some may be hyper-sensitized to witnessing violence against people they know. Untreated trauma complicates the developmental trajectory for young people, and evidence now shows that many mental illnesses begin in adolescence. Demographic factors and the severity and chronicity of exposures for youth exposed to violence are associated with numerous other symptoms and forms of psychopathology [56, 57], as well as suicidal and self-injurious behavior [58].

These findings have implications for research and practice. As a matter of practice, young people should be screened for pre-existing trauma exposure and associated symptoms before receiving a mental health diagnosis. Trauma science now shows that trauma-based conditions are disorders of affect or ‘emotion’ (anxiety, irritability, depression) and regulation. Youth of the age in our sample are often subject to harsh school disciplinary practices if they display behavioral regulation challenges in the classroom. ‘Dysregulated’ students (a common diagnosis is, for example, Attention Deficit Hyperactivity Disorder, or ADHD) are typically treated with cognitive behavioral therapy and/or medications, rather than trauma-focused treatment activities. As more schools and youth-serving institutions aspire to be trauma-informed and trauma-responsive, interventions must address the needs of youth who display both internalizing and externalizing behaviors, and who have experiences of acute and chronic exposures to traumatic events. Young people with chronic stressors such as poverty, discrimination and ongoing exposures to violence should have access to a variety of treatment activities and be a part of longitudinal studies. In communities where lower SES is associated with increased exposures to indirect violence and all types of adverse MH symptomatology, effective interventions must go beyond individualized treatment and take more collective and small group approaches to trauma response.

## Supporting information

S1 Table Emotional wellness screener item extracts. (DOCX)

S2 Table Indirect exposure to violence and adverse MH Symptomatology by grade grouping. (DOCX)

S1 Dataset (XLSX)

S1 Codebook (XLSX)
