# Supplementary material for: Indirect violence exposure and mental health symptoms among an urban public-school population: Prevalence and correlates
Source: PLoS One. 2019 Nov 27;14(11):e0224499. doi: 10.1371/journal.pone.0224499 (PMC6881142; doi:10.1371/journal.pone.0224499)
Supplement: S1 Table — (DOCX) [file pone.0224499.s001.docx]

**S1 Table. Selected Emotional Wellness Screener Measures**

| Item | Label | Question |
| --- | --- | --- |
| PTSD Screener | | |
| *In your life, have you ever been in a situation that was so frightening, horrible, or upsetting that a month later, you:* | | |
| 1 | *Bad Dreams* | Had bad dreams or nightmares? |
| 2 | *Avoidance* | Tried hard not to think about it, talk about it, or have feelings about it? |
| 3 | *Jumpy* | Felt jumpy or easily startled or felt that your world was unsafe and you had to protect yourself? |
| 4 | *No Strong Feelings* | Were not able to have strong feelings, for example, not able to cry or not able to feel happy? |
| 5 | *Irritable* | Felt irritable or had fits of anger – got mad easily? |
| 6 | *Bad Thoughts - Self* | Had bad thoughts about yourself? |
| 7 | *Bad Thoughts - Others* | Had bad thoughts about others? |
| 8 | *Bad Thoughts - Environment* | Had bad thoughts about your environment? |
| 9 | *4+ Experiences in Past Month* | Have you had 4 or more of these experiences during the past month? |
| Depression Screener | | |
| *In the past two weeks:* | | |
| 10 | *Sad* | Have you felt depressed (sad, blue) most days? |
| 11 | *Lost Interest* | Have you lost interest in things and/or people that you used to enjoy? |
| 12 | *Worried* | Have you been worried most days? |
| 13 | *Problems Concentrating* | Have you had problems concentrating? |
| 14 | *Change in Appetite* | Have you noticed a change in your appetite—eating too little or too much? |
| 15 | *Change in Sleep Pattern* | Have you noticed a change in your sleeping pattern—too little or too much sleep? |
| 16 | *Substance Use* | Have you used alcohol, drugs or weed to help you feel better about yourself or fit in? |
| 17 | *Suicide Ideation* | Have you thought about killing yourself? |
| Indirect Violence Exposure | | |
| *At any time in your life:* | | |
| 18 | *Witnessed Violence Against Parent* | Did you SEE a parent get pushed, slapped, hit, punched, or beaten by another parent or their boyfriend/girlfriend? |
| 19 | *Witnessed Violence Against Sibling* | Did you SEE your parent push, slap, hit, punch, or beat your brothers or sisters? (not including a spanking)? |
| 20 | *Witnessed Shooting/Stabbing/Beating* | Did you SEE anyone get shot, stabbed, or beaten in real life? (not including video games/TV/movies)? |
| 21 | *Witnessed Murder* | Did you SEE someone murdered in real life? (not including video games/TV/movies) |
| 22 | *Murder of Someone Close* | Was someone close to you murdered, like a friend, neighbor, classmate or someone in your family? |
