# Supplementary material for: Indirect violence exposure and mental health symptoms among an urban public-school population: Prevalence and correlates
Source: PLoS One. 2019 Nov 27;14(11):e0224499. doi: 10.1371/journal.pone.0224499 (PMC6881142; doi:10.1371/journal.pone.0224499)
Supplement: S2 Table — (DOCX) [file pone.0224499.s002.docx]

**S2 Table. Indirect Exposure to Violence and Adverse Mental Health Symptomatology by Grade Grouping (N=1548).**

| Measure | <9^th^ Grade  n (%) | ≥9^th^ Grade  n (%) | Cramer’s V | p-value |
| --- | --- | --- | --- | --- |
| Witnessed parent get pushed, slapped, hit, punched, or beaten by another parent or parent’s partner | 276 (28.87) | 180 (32.85) | 0.0416 | 0.1064 |
| Witnessed sibling get pushed, slapped, hit, punched, or beaten by parent (not including spanking) | 151 (15.71) | 98 (17.88) | 0.0281 | 0.2747 |
| Witnessed shooting/stabbing/beating | 380 (39.24) | 260 (47.27) | 0.0772 | 0.0026 |
| Witnessed murder | 164 (17.10) | 118 (21.49) | 0.0542 | 0.0353 |
| Experienced the murder of someone close | 518 (53.90) | 306 (55.74) | 0.0177 | 0.4908 |
| Positive screening for depression | 197 (20.21) | 130 (23.38) | 0.0373 | 0.1448 |
| Positive screening for lifetime PTSD | 462 (47.43) | 238 (42.88) | -0.0439 | 0.0859 |
| Positive screening for current PTSD | 279 (28.64) | 132 (23.78) | -0.0527 | 0.0392 |
